# An experimental test on the effects of dispersal from different habitat sources on community structure

**DOI:** 10.1002/ecy.70256

**Published:** 2025-11-17

**Authors:** Gustavo L. Villarreal, Fernanda A. S. Cassemiro, Priscilla Carvalho, Luis M. Bini, Jascieli C. Bortolini, Amanda C. F. Queiroz, Wilson M. Leão‐Neto, Roger P. Mormul, Ludgero C. G. Vieira, João C. Nabout, Fabricio B. Teresa, Maisa C. Vieira, Karine B. Machado, Tadeu Siqueira, Adriano S. Melo

**Affiliations:** ^1^ Programa de Pós‐Graduação em Ecologia & Evolução Universidade Federal de Goiás, Campus Samambaia Goiânia Goiás Brazil; ^2^ Departamento de Ecologia, Instituto de Ciências Biológicas Universidade Federal de Goiás, Campus Samambaia Goiânia Goiás Brazil; ^3^ Departamento de Botânica, Instituto de Ciências Biológicas Universidade Federal de Goiás, Campus Samambaia Goiânia Goiás Brazil; ^4^ Programa de Pós‐Graduação em Ecologia e Conservação da Biodiversidade, Instituto de Biociências Universidade Federal do Mato Grosso Cuiabá Mato Grosso Brazil; ^5^ Departamento de Biologia, Centro de Ciências Biológicas Universidade Estadual de Maringá Maringá Paraná Brazil; ^6^ Núcleo de Estudos e Pesquisas Ambientais e Limnológicas (NEPAL) Universidade de Brasília, Campus Planaltina Planaltina Federal District Brazil; ^7^ Instituto Acadêmico de Ciências da Saúde e Biológicas Universidade Estadual de Goiás Anápolis Goiás Brazil; ^8^ Instituto Acadêmico de Ciências da Saúde e Biológicas Universidade Estadual de Goiás, Unidade Universitária de Porangatu Porangatu Goiás Brazil; ^9^ Instituto de Biociências Universidade Estadual Paulista Rio Claro São Paulo Brazil; ^10^ School of Biological Sciences University of Canterbury Christchurch New Zealand; ^11^ Departamento de Ecologia, Instituto de Biociências Universidade Federal do Rio Grande do Sul Porto Alegre Rio Grande do Sul Brazil

**Keywords:** beta diversity, ecological drift, mass effect, mesocosm, metacommunity, rescue effect, zooplankton

## Abstract

Biological interactions, disturbances, and demographic stochasticity often drive population declines and local extinctions. Dispersal can counterbalance these drivers by rescuing small populations or facilitating recolonization. Using freshwater zooplankton in experimental mesocosms, we tested three hypotheses: (1) isolated sites would experience declines in species richness, with ecological drift causing communities to lose different species and become more dissimilar over time; (2) communities connected by dispersal from similar habitats would maintain their species richness and composition, as arriving species balance losses through rescue effects and recolonization, thereby halting community differentiation; and (3) dispersers originating from different sources may establish themselves in recipient communities through mass effects, resulting in higher species richness compared to communities receiving dispersers from similar habitat sources. Thirty 500‐L tanks were initially colonized with zooplankton from lake A, and 10 tanks with colonizers from lake B, which had partially distinct species composition. Tanks were kept isolated for 50 days, after which 10 tanks initially colonized by lake A began receiving dispersers from paired tanks also colonized by lake A (treatment Aa). Another 10 tanks colonized by lake A received dispersers from paired tanks colonized by lake B (Ab). We found that isolated communities (A0, B0) tended to lose species over time and differentiate from one another, indicating differential local extinctions. Communities receiving dispersers from the same habitat (Aa) halted species losses and maintained their species richness, whereas those receiving species from a different habitat (Ab) not only halted species losses but also accumulated additional species over time. Treatments receiving dispersers (Aa, Ab) exhibited beta diversity (among replicates within treatments) similar to levels observed prior to dispersal events. Comparisons of paired source‐recipient tanks (A0–Aa, B0–Ab) further supported the finding of differential extinctions in isolated communities. Our results demonstrate that dispersal counteracts declining species richness and increasing differentiation caused by differential local extinctions in isolated communities, either through rescue or mass effects.

## INTRODUCTION

Dispersal is a key mechanism determining the species composition of local communities (Leibold & Chase, 2018). For instance, small fragments or islands harbor fewer species than larger areas, often favoring those with higher dispersal abilities (Wang et al., 2024). Dispersal also plays a fundamental role in maintaining local diversity, as it can counterbalance local extinctions through recolonization (Brown & Kodric‐Brown, 1977). Furthermore, dispersal can mitigate anthropogenic or natural disturbances (Limberger et al., 2019), enhancing community resilience and helping to maintain ecosystem functions (Harvey et al., 2020).

Local communities are rarely fully isolated; even oceanic islands receive dispersers. However, ecological theory predicts that isolated communities will lose species over time due to interspecific competition, demographic stochasticity, and weak stabilizing forces (Leibold et al., 2004; Lerch et al., 2023; Ron et al., 2018; Vellend, 2010). While theory suggests predation, disturbances and habitat heterogeneity can promote coexistence by regulating dominant competitors (Gurevitch et al., 2000) or creating niche opportunities (Mouquet & Loreau, 2003), in practice these mechanisms are not fully sufficient or consistently active to prevent local extinctions. Consequently, gradual local extinctions occur, leading to divergent compositional trajectories across more isolated communities. Demographic drift and subtle differences in environmental conditions, resource availability, and initial species composition can cause small local communities to lose different species or shift dominance patterns (Cadotte, 2006; Le Moigne et al., 2023; Werner et al., 2020). Over time, this leads to declining local (alpha) diversity but increasing beta diversity among isolated communities, as ecological selection and drift amplify compositional dissimilarity (Chase, 2003; Gilbert & Levine, 2017; Vellend, 2010).

Dispersal rate can have contrasting effects on recipient communities (Lerch et al., 2023; Mouquet & Loreau, 2003). A low to moderate influx of dispersers from source communities of the same habitat can offset extinctions driven by selection and drift by increasing population sizes (rescue effect; Brown & Kodric‐Brown, 1977). Because these dispersers come from the same local species pool (Zobel, 1997), they often reintroduce species that were previously lost, thereby maintaining local diversity. When multiple communities exchange dispersers at such rates, beta diversity remains constant over time. In contrast, high dispersal rates may cause local species richness to converge with that of the metacommunity (Loke & Chisholm, 2023), reducing beta diversity as species become uniformly distributed across local communities (Mouquet & Loreau, 2003; Ron et al., 2018). Moreover, increased dispersal can enlarge the effective community size, reducing the impact of demographic drift and strengthening selection, ultimately favoring superior competitors across communities (Cadotte, 2006; Forbes & Chase, 2002; Mouquet & Loreau, 2003; Ron et al., 2018).

The effects of dispersal among local communities depend on the species identities of the dispersers. Species arriving from source communities with different environmental conditions may struggle to establish viable populations in the long term. However, if dispersal from source (Pulliam, 1988) or core communities (Shmida & Wilson, 1985) occurs frequently enough, these species may persist in the recipient (sink) community despite being poorly adapted to local conditions (Limberger et al., 2019). Sink communities are likely to support smaller populations of these maladapted species than source communities of similar size (Pulliam, 1988). Nevertheless, their arrival increases species richness in recipient communities. Empirical studies show that species‐rich communities are often composed largely of rare species, many of which are abundant in other habitats (Sgarbi & Melo, 2018). In contrast, dispersal between source and recipient communities with similar environmental conditions, or the same local species pool (Zobel, 1997), does not have this effect. The effect of new arrivals on beta diversity among recipient communities also depends on how heterogenous source communities are (Voelker & Swan, 2021). When beta diversity among source communities is high, recipient communities receive different sets of species, thus increasing beta diversity among them (Fukami, 2015; Shmida & Wilson, 1985). Conversely, when beta diversity among source communities is low, dispersal has little effect on the spatial beta diversity of recipient communities.

**FIGURE 1:**
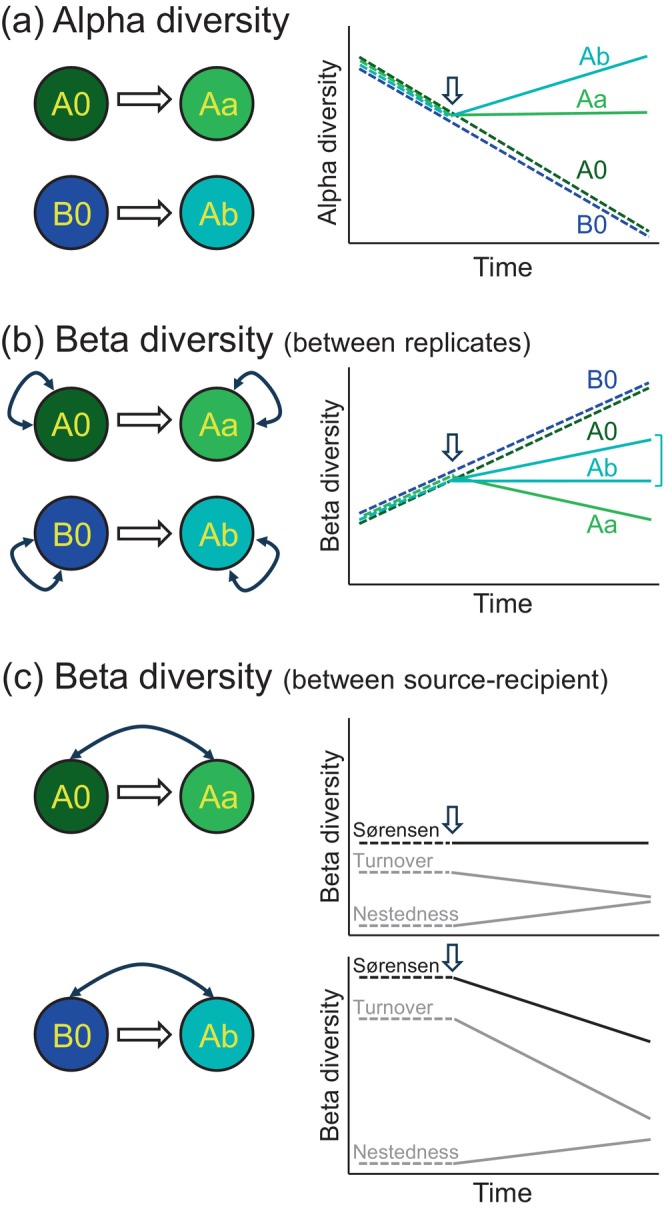
Experimental communities with different dispersal treatments and predictions for alpha and beta diversities. Open arrows indicate unidirectional dispersal. A0 and Aa received water from lake A, but A0 did not receive dispersal, whereas Aa received dispersal from a paired A0 community. B0 received water from lake B and did not receive dispersal. Ab was filled with water from lake A but received dispersal from a paired B0 community. Solid arrows indicate beta diversity comparisons. Dashed lines indicate treatments without dispersal (A0, B0) or periods before dispersal events.

We assessed the effects of dispersal on alpha and beta diversity in experimental zooplankton communities. To do so, we assembled 40 water tanks (500 L each) and established replicated communities—either connected by unidirectional dispersal or isolated—to generate three scenarios for testing our hypothesized causal mechanisms. We predicted that in the absence of dispersal (treatments A0 and B0 in Figure 1), ecological drift would dominate, leading to a decline in species richness over time (Figure 1a). Beta diversity among local communities within each isolated treatment should increase over time as different species would be lost in each independent community (Figure 1b; A0–A0 and B0–B0 comparisons). In a scenario where dispersal occurs from local communities within the same habitat (low beta diversity or the same local species pool; treatment Aa in Figure 1), we expected the rescue effect and recolonization to offset ecological drift, maintaining species richness (Figure 1a). Beta diversity would decline over time as recolonization and rescue from the same habitat would homogenize species composition across communities (Figure 1b, Aa–Aa comparison). In the final scenario, where dispersal occurs from local communities of a different habitat (high beta diversity or different local species pool; Ab in Figure 1), we expected ecological drift to be counterbalanced by a combination of recolonization, rescue and mass effects, leading to increased species richness (Figure 1a). Mass effects should be caused by the arrival of species that were initially absent. Even if some of these species are poorly adapted to the new conditions, their persistence would be supported by repeated dispersal. Dispersal should maintain or slightly increase beta diversity, as different species would persist across recipient communities (Figure 1b, Ab–Ab comparisons).

We further investigate how dispersal changed the species composition of connected communities. This was done by examining the turnover and nestedness components of beta diversity of connected communities compared with their isolated source communities. Turnover between an isolated community (A0) and an initially similar community receiving dispersers from the same habitat (Aa) is expected to be low and to be maintained over time, as both communities derived from the same habitat and share the same local species pool (Figure 1c, A0–Aa comparison). Nestedness should increase over time as species richness in the dispersal‐receiving treatment (Aa) is expected to remain stable, while the isolated communities (A0) should lose species. By contrast, beta diversity between local communities B0 and Ab should be high initially, given their different habitat origins. Dispersal from B0 to Ab should reduce beta diversity, as turnover should decrease as Ab accumulates species from B0. This reduction, however, would be at least partially counterbalanced by an increase in nestedness as B0 loses species.

## METHODS

### Experimental setup

The experiment used 40 circular water tanks of 500 L spaced 1.5 m apart (Appendix S1: Figure S1). We selected two lakes with different limnological characteristics and aquatic communities to partially fill the water tanks and set up the treatments. The first lake, located in Goiânia (16°35′38″ S, 49°16′50″ W), is 43 km from the experimental area (lake A hereafter). It lies on the Universidade Federal de Goiás campus and is used for scientific experiments and to supply water to adjacent experimental ponds. The second lake, located at Fazenda Santa Branca in Teresópolis de Goiás (16°25′07″ S, 49°05′28″ W), is 16 km from the experimental area (lake B hereafter) and is used for ecotourism and fishing. Both lakes receive domestic and agricultural effluents but did not present eutrophic conditions.

Initially, tanks were filled with 200 L of water from their respective lakes (30 tanks with water from lake A and 10 tanks with water from lake B). Water was collected using a small bilge pump. The remaining 300 L of water to fill each tank was obtained from a local well. Qualitative sampling of the well revealed 38 zooplankton species, including testate amoeba, Rotifera, Cladocera, and Copepoda, some of which were also present in the two lakes. The effect of these species was identical across all tanks and thus did not contribute to treatment differences. Water from the well was added to compensate for evaporation. For treatments A0 and B0, we also added water to replace the volumes transferred to their paired tanks to establish the dispersal treatments (see below). Nutrients were added to tanks to match levels observed in the lakes (Appendix S1).

Water tanks were covered with a semi‐transparent mesh (1.4 × 0.7 mm) to exclude terrestrial animals from entering (Appendix S1: Figure S1). The cover can also reduce unintentional dispersal among tanks. In the event of unintended dispersal, the random assignment of tanks to treatments ensures that any such effects are evenly distributed across treatments (Appendix S1: Figure S2). Water in tanks was mixed weekly, and samples were always taken after mixing.

### Dispersal events

We implemented two treatments that did not receive dispersers, differing only in water origin: one derived from lake A (treatment A0) and the other from lake B (treatment B0; Figure 1). After 50 days, we introduced two other treatments in tanks originally filled with water from lake A, which began receiving dispersers from a paired local community. Dispersal events were conducted unidirectionally by transferring 5 L (1% of the total 500 L volume) of water from tanks in treatments A0 and B0 into separated tanks with lake A water, generating treatments Aa and Ab, respectively (Figure 1). The dispersal volume was chosen to be large enough to simulate the hypothesized mechanisms (rescue and mass effects) but not so large as to homogenize the communities across water tanks (Vogt & Beisner, 2011). Thus, in treatment Aa, dispersers originated from the same habitat (treatment A0), whereas in treatment Ab, dispersers came from a different habitat (treatment B0). Each of the four treatments (A0, B0, Aa, and Ab) was replicated in 10 tanks. We sampled tanks on days 20, 50, 65, 86, and 101 after the experiment was set up. Pulse dispersal events occurred between paired source‐recipient tanks on days 50, 65, and 86, immediately after sample collection. In total, 200 samples were obtained (40 tanks × 5 sampling events).

### Sampling and identification

Samples consisted of 25 L (5% of the volume of tanks) filtered through a 40 μm mesh plankton net. Samples were stored in 300 mL polyethylene bottles and fixed with buffered formaldehyde solution. In the laboratory, samples were filtered through a 40 μm mesh and then diluted in 50 mL of distilled water. Aliquots were taken with a Hensen‐Stempel pipette and examined under an optical microscope using Sedgwick‐Rafter chambers. The entire sample was processed, and organisms were identified to the lowest possible taxonomic level (usually species) using specialized taxonomic keys (references in Lansac‐Tôha et al., 2009). Non‐adult copepods (nauplii and copepodites) could not be identified and were not included in the analyses.

### Data analyses

We rarefied samples to standardize sampling effort to 126 individuals, the minimum abundance observed across all 200 samples, using the function vegan::rarefy (Oksanen et al., 2024) in the R environment (R Core Team, 2024). Observed and rarefied species richness were highly correlated (Pearson's *r* = 0.997), as few species were rare (the rarest species had 38 individuals). Thus, we used observed species richness in analyses. We ran a repeated‐measures ANOVA (rm‐ANOVA) to test the effects of treatments (4 levels), sampling dates (5 levels), and their interaction on species richness using the R function ez::ezANOVA (Lawrence, 2016). The sphericity assumption was violated (Mauchly's test, W = 0.442, *p* < 0.001), so *p*‐values were adjusted using the Greenhouse–Geisser procedure.

Multivariate exploratory analyses were done using Nonmetric multidimensional scaling (NMDS) with Sørensen (presence‐absence) and Bray–Curtis (abundance‐based) dissimilarities; abundance data were log(*x* + 1) transformed. Both ordinations included all 200 samples, but results are shown separately by sampling date for clarity. Differences in community composition and structure were assessed using permutational multivariate ANOVA (PERMANOVA) (Anderson, 2001). The ordination and the PERMANOVA were done using the R functions vegan::metaMDS and vegan::adonis2, respectively. Pair‐wise PERMANOVA comparisons were done using pairwiseAdonis::pairwise.adonis2 (Arbizu, 2017).

We assessed beta diversity within‐ and between‐treatments at each sampling date. For within‐treatments assessment, we tested whether beta diversity changed over time using permutational analysis of multivariate dispersions (PERMDISP; Anderson et al., 2006), as implemented in the R function vegan::betadisper. This analysis calculates distances of samples to their groups' (i.e., each of the five sampling events) median in a Principal Coordinate space, ensuring each sample contributed only once to avoid pseudoreplication. Analyses were done separately for each treatment using the Sørensen and Bray–Curtis (log(abundance + 1)) dissimilarity matrices.

For the between‐treatments comparisons, beta diversity was calculated between paired source‐recipient water tanks according to the experimental design. We partitioned Sørensen dissimilarity into turnover (Simpson index; sim hereafter) and nestedness (sne hereafter) components (Baselga, 2010) using the R function betapart::beta.pair (Baselga et al., 2018). The NMDS and PERMDISP analyses yielded similar results for presence‐absence and abundance data, so only presence‐absence data were used for the source‐recipient comparisons. Dissimilarities were calculated using experimental source‐recipient pairs, ensuring each sample was used once per sampling event.

We used Beta Regression (Ferrari & Cribari‐Neto, 2004) to analyze the dissimilarities between donating and receiving tank pairs, thereby testing the effects of dispersal sources (A0 and B0) on recipient communities (Aa and Ab, respectively) across sampling events. The model included dissimilarity as the response and factors for dispersal source (two levels: A0–Aa, B0–Ab) and sampling event (five levels: T1–T5) and their interaction. A significant interaction (see *Results*) prompted separate models for each dispersal source. Dispersal effects were assessed by comparing the sampling event immediately before dispersal (T2) with the initial sampling (T1) and subsequent sampling events. Beta Regression was performed using glmmTMB::glmmTMB, with likelihood ratio tests (lmtest::lrtest; Zeileis & Hothorn, 2002) and post‐hoc contrasts (emmeans::lsmeans, emmeans::contrast; Lenth, 2024).

## RESULTS

Across the 200 samples, we recorded 88,774 individuals, with a mean abundance of 443.9 individuals (range: 126–1225) per sample of 25 L (mean of 17.8 and range of 5.0–49.0 individuals/L). We recorded 63 species from four taxonomic groups: 23 testate amoeba, 17 Rotifera, 14 Cladocera, and 9 Copepoda. Species richness per sample averaged 19.4 (range: 9–30). The rarest species, *Daphnia gessneri* (Cladocera), was represented by 38 individuals, while the most common species, *Notodiaptomus brandorffi* (Copepoda), accounted for 4792 individuals. Prior to dispersal events (sampling events T1 and T2), the 80 samples collected from all tanks contained 63 species. As expected, compositional differences were evident between tanks initially colonized by lakes A and B. Tanks with water from lake A (treatments A0, Aa, and Ab; *n* = 60 samples at T1 and T2) included 38 species, while tanks filled with water from lake B (treatment B0; *n* = 20 samples at T1 and T2) contained 37 species. Of these, 12 species were shared between the two sets of tanks, while 26 and 25 species were exclusive to tanks filled with water from lakes A and B, respectively.

**FIGURE 2:**
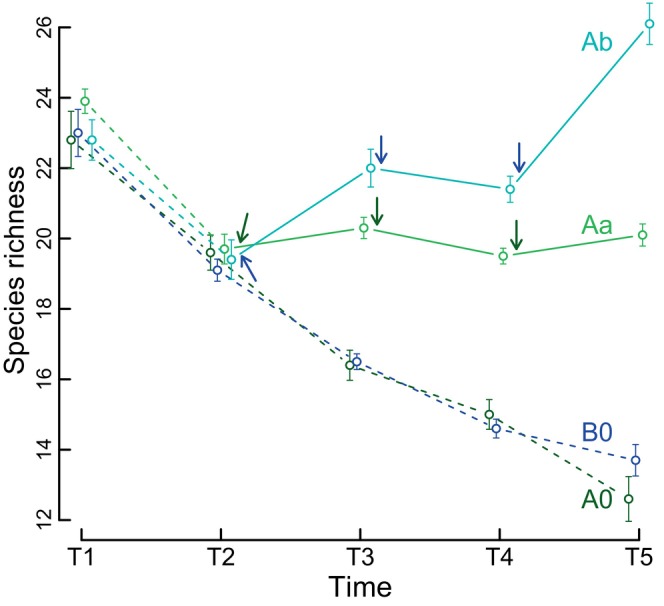
Mean species richness (±SE) in experimental communities subjected to four treatments. T1, T2, T3, T4, and T5 refer to samplings, respectively, on days 20, 50, 65, 86, and 101 after the setup of the experiment. Arrows indicate three dispersal events, performed immediately after samples were obtained. Dashed lines indicate treatments without dispersal (A0, B0) or periods before dispersal events (T1–T2 for Aa and Ab).

The effects of treatments on species richness depended on time (interaction term: *F*_12,144_ = 34.75, *p* < 0.001; Figure 2). At the first sampling event (T1: 20 days after tanks were filled), species richness was similar across all treatments. By the second sampling event (T2: 50 days after filling), species richness had decreased similarly across all treatments. This decline continued until the end of the experiment in tanks that did not receive dispersers (A0 and B0), with these tanks harboring roughly half the species present at T1 by the final sampling event (T5: day 101). By contrast, the decreasing trend in species richness observed between T1 and T2 was interrupted in tanks that received dispersers from tanks with the same water origin (Aa; Figure 2). By the end of the experiment (101 days after tanks were filled), species richness in Aa tanks was similar to levels observed before the first dispersal event (T2). In treatment Ab, dispersal from paired tanks with a different water origin (B0) halted the initial decline in species richness and led to a subsequent increase (Figure 2).

**FIGURE 3:**
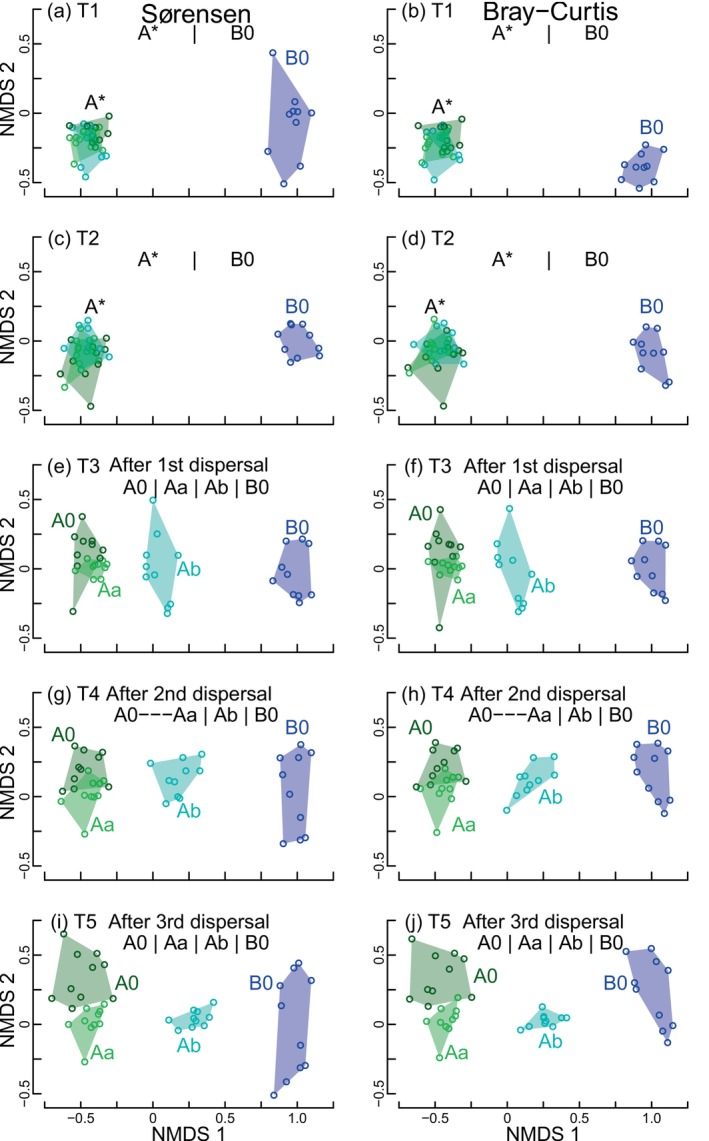
Nonmetric multidimensional scaling (NMDS) using presence/absence (Sørensen dissimilarity) and abundance (log[*x* + 1]) (Bray–Curtis dissimilarity) data for each sampling time. T1–T5 refers to sampling on days 20, 50, 65, 86, and 101 after the experiment setup. Samples at T1 and T2 (a–d) were not subjected to dispersal, and blue symbols ordinated at the right‐hand side indicate those from lake B whereas the remaining ones from lake A (labeled as A*; they are plotted with different colors to reflect the dispersal treatments they will receive). Samples at T3, T4 and T5 (e–j) were obtained after the first, second and third dispersal events, respectively, and show the differentiation of tanks filled with water from lake A that received dispersal from tanks filled with water from lake B (Ab). A0 (dark green) and B0 (dark blue) indicate tanks that were not subjected to dispersal. Tanks Aa (light green) were filled with water from lake A and received dispersal of a paired tank also filled with water from the same lake. Vertical bars indicate differences (*p* < 0.05) and dashes similarities (*p* > 0.05) in species composition (Sørensen) or community structure (Bray–Curtis) assessed using pair‐wise permutational multivariate ANOVA (PERMANOVA).

Results of the NMDS and PERMANOVA analyses were consistent using presence‐absence and abundance data (Figure 3). Before dispersal events (T1 and T2), tanks filled with water from lake A (A0, Aa, and Ab) were compositionally similar to one another but different from those filled with water from lake B (B0) (four tests: T1 and T2 and using presence‐absence and abundance data; *F*_1,38_ > 70.0, *p* < 0.001 in all cases; Figure 3a–d). Following the first dispersal event (65 days after tanks were filled), tanks receiving dispersers from paired tanks colonized with lake B water (Ab) occupied intermediate positions along the first ordination axis between the A0–Aa and B0 groups (*F*_3,36_ > 18.1, *p* < 0.001 either for presence‐absence or abundance data; Figure 3e,f). After the second and third dispersal events (86 and 101 days after tanks were filled, respectively), Ab tanks shifted further toward B0 tanks (*F*_3,36_ > 16.1, *p* < 0.001 for T4 and T5 and using presence‐absence and abundance data; Figure 3g–j). Tanks receiving dispersers from paired tanks filled with the same water origin (Aa) showed slight compositional differences (lower scores on axis 2) compared with isolated tanks with the same water origin (A0) (Figure 3). This shift reflected the decrease in species richness observed in A0 tanks (Figure 2).

**FIGURE 4:**
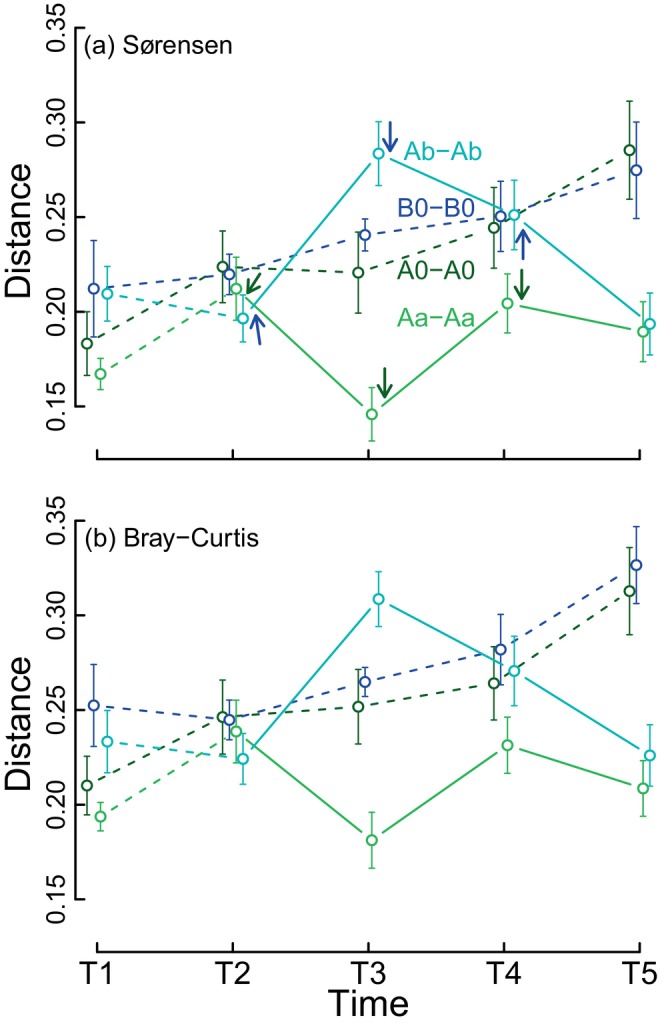
Temporal variation in beta diversity (among the tanks of the same treatment) given by the mean (±SE) distance of replicates to the group spatial median in a Principal Coordinate Ordination.

Within‐treatment beta diversity (among the 10 replicates) varied over time but depended on treatment (Figure 4). Results were consistent for presence‐absence and abundance (Figure 4a,b). Beta diversity increased linearly and at similar rates for the two treatments that did not receive dispersers (PERMDISP analysis; A0: Sørensen: *F*_4,45_ = 3.13, *p* = 0.024; Bray–Curtis: *F*_4,45_ = 3.60, *p* = 0.013; B0: Sørensen: *F*_4,45_ = 1.71, *p* = 0.165; Bray–Curtis: *F*_4,45_ = 3.79, *p* = 0.010). The first dispersal event homogenized tanks in the treatment Aa (Sørensen: *F*_4,45_ = 3.59, *p* = 0.013; Bray–Curtis: *F*_4,45_ = 3.01, *p* = 0.028; Figure 4), but increased differentiation among tanks in treatment Ab (Sørensen: *F*_4,45_ = 6.12, *p* < 0.001; Bray–Curtis: *F*_4,45_ = 5.30, *p* = 0.001; Figure 4). However, these patterns were not maintained in subsequent dispersal events. By the final sampling events, beta diversity in both Aa and Ab treatments returned to levels similar to those observed before dispersal (Figures 3i,j and 4).

**FIGURE 5:**
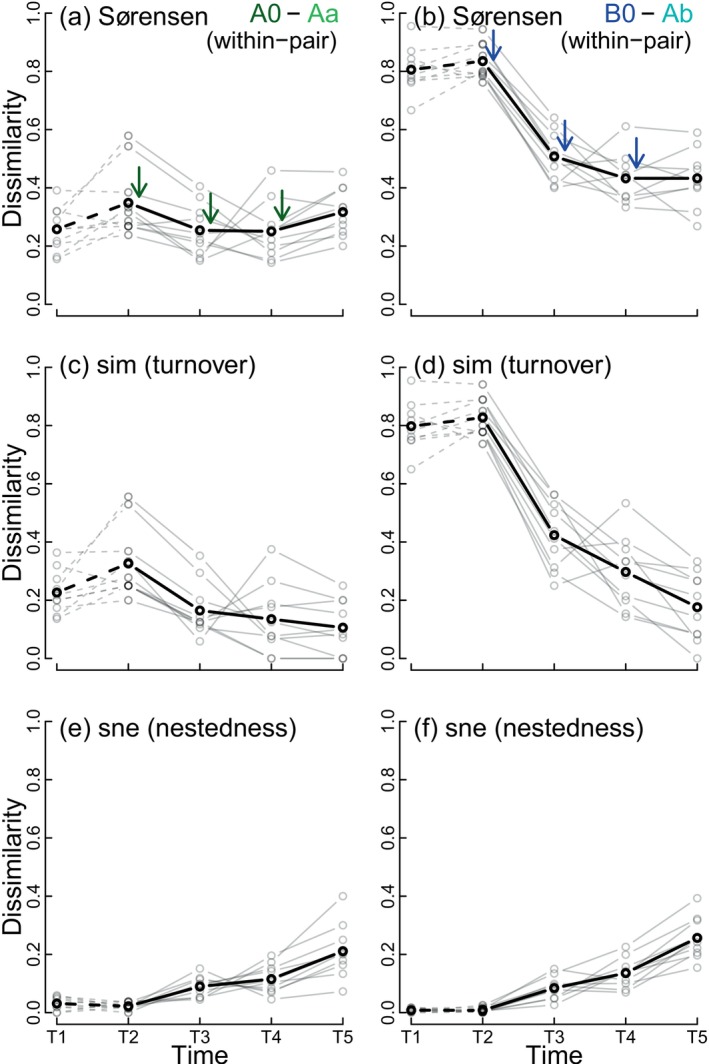
Within‐pair Sørensen dissimilarity (a, b) and its turnover (c, d) and nestedness (e, f) components between tanks connected by dispersal sampled at five dates. Black lines indicate averages, and gray lines the 10 paired tanks A0–Aa (a, c, e) and B0–Ab (b, d, f).

Differences in source‐recipient beta diversity between A0–Aa and B0–Ab pairs varied over time (Beta Regression, interaction term, χ^2^ = 67.90, df = 4, *p* < 0.001; Figure 5a,b). Dissimilarities were generally higher for B0–Ab than for A0–Aa, particularly before dispersal events. For A0–Aa pairs, Sørensen dissimilarity varied slightly over time (χ^2^ = 10.51, df = 4, *p* = 0.033; Figure 5a). Comparisons with sampling event T2 showed no difference for event T1 (*p* = 0.051). However, significant differences were observed after dispersal for events T3 (*p* = 0.035) and T4 (*p* = 0.022), with dissimilarities returning to predispersal levels by T5 (*p* = 0.790).

In contrast, dissimilarity for B0–Ab pairs was high before dispersal events (reflecting their initial colonization by water from different lakes) but decreased sharply after dispersal (χ^2^ = 87.55, df = 4, *p* < 0.001; Figure 5b). Comparisons to T2 showed no difference for T1 (*p* = 0.790) but significant differences for all post‐dispersal events (in all three cases *p* < 0.001).

Interestingly, the two components of Sørensen dissimilarity for the source‐recipient comparison exhibited opposing temporal trends (Figure 5, comparisons c–e and d–f). For A0–Aa pairs, the low temporal variability in total dissimilarity resulted from a decrease in turnover (Figure 5c) coupled with an increase in dissimilarity due to nestedness (Figure 5e). Similarly, B0–Ab pairs showed opposite temporal trends in turnover and nestedness (Figure 5d,f), although the strong decrease in turnover (Figure 5d) was only partially compensated by the moderate increase of the nested‐based dissimilarity (Figure 5f).

## DISCUSSION

Our predictions about the effects of dispersal on community composition were largely confirmed. Dispersal halted the species loss observed in isolated communities and resulted in increased species richness when dispersers came from a different local species pool. Species composition in isolated communities tended to differentiate over time, whereas dispersal caused communities to maintain their similarities. Dispersal caused communities to exhibit a decrease in turnover but an increase in nestedness over time, particularly when dispersers originated from a different local species pool.

Isolated communities experienced many local extinction events, leading to reduced species richness by the end of the experiment. This aligns with patterns observed in isolated habitats like oceanic islands, fragmented landscapes, and headwater streams (e.g., Carrara et al., 2012). Local species richness reflects a balance between colonization and extinction (e.g., Brown & Kodric‐Brown, 1977), with extinctions driven by biotic interactions, low adaptation to local conditions, and demographic stochasticity due to small population sizes (Drake, 1991; Fukami, 2015). In our study, environmental differences between tanks and the source lake (e.g., temperature fluctuations, water‐sediment dynamics, and resting‐egg bank) likely exacerbated these factors, particularly given the small initial population sizes in tanks (Siqueira et al., 2020). Many species required dispersal to persist, as seen in communities that received dispersers from the same habitat (A0 → Aa). The fast impact of dispersal on recipient communities was surprising, given the small volume of dispersers (5 L, 1% of recipient volume) and the species‐poor source (A0). Dispersal from a different habitat (B0 → Ab) not only allowed the recolonization of locally extinct species but also introduced new species, increasing richness over time. Despite moderate compositional similarity between source lakes (~1/3 shared species), local conditions in recipient communities allowed colonization of previously absent species. This led to species accumulation in communities receiving dispersers from different habitats, consistent with our prediction based on source‐sink dynamics (Chase, 2003; Mouquet & Loreau, 2003).

Although isolated communities lost species over time, our analysis indicated divergent compositional trajectories, with different tanks losing different species due to high stochasticity in extinctions. Despite identical initial resources and conditions, minor compositional differences likely allowed historical contingencies (Drake, 1991; Fukami, 2015; Le Moigne et al., 2023) to drive variation in species occurrences and relative abundances. Low inoculation densities (200 L of source lake water) and small tank volumes (500 L) relative to source lakes may have further amplified these contingencies, as small initial populations are prone to priority effects (Drake, 1991; Fukami, 2004, 2015). Similar stochastic species loss has been observed in laboratory experiments with dispersal limitation (Carrara et al., 2012; Le Moigne et al., 2023). Interestingly, the differentiation of isolated communities over time resembles patterns in naturally colonized experimental ponds (Jenkins & Buikema, 1998), although driven by different processes. In our study, variation arose primarily from differential extinction, whereas Jenkins and Buikema (1998) attributed variation to differential colonization. Beyond stochastic colonization and extinction, additional variation can stem from priority effects, where early arriving species affect the establishment of late arriving species (Chase, 2003; Drake, 1991; Fukami, 2015). Similarly, Torres et al. (2024) suggested that inverse priority effects, where stochastic extinctions differently impact remaining species, may contribute to variation, as observed in our isolated communities. Thus, alongside local factors, natural community variation likely results from stochastic colonization–extinction dynamics and their associated priority and inverse priority effects.

Dispersal, whether from the same or different habitats, reduced beta diversity among replicates (within treatments) by the end of the experiment compared to isolated treatments, aligning with our expectations (Chase, 2003; Mouquet & Loreau, 2003). However, the trajectories of these changes differed depending on the source community. Dispersal from the same habitat caused a sharp decline in beta diversity after the first dispersal event, likely due to the homogenizing effect of recolonization by species that had been extirpated locally. However, the establishment of newly arriving species depended on recipient community structure. As in the isolated treatments, communities lost different sets of species, leading to variation in composition and relative abundances among recipient communities. Thus, the initial reduction in beta diversity partially reversed in later sampling events, as many dispersers failed to establish viable populations. Nevertheless, some species successfully colonized recipient communities, resulting in beta diversity (Ab or Aa) at the last sampling event to be much lower than in isolated communities (A0 or B0).

The temporal trajectory of beta diversity in communities receiving dispersers from a different habitat peaked after the first dispersal event, likely due to two processes: distinct disperser sets from source communities and varying establishment success in recipient communities. Over time, isolated donor communities differentiated, providing unique disperser sets. Recipient communities, already distinct from each other before dispersal, provided varied biotic resistances that influenced establishment success, allowing some species to colonize while others failed (Drake, 1991). Consequently, many dispersers could not maintain viable populations in recipient communities, and final beta diversity among replicates returned to intermediate values, similar to communities receiving dispersers from the same habitat. Previous studies suggest colonization success depends on resident species composition (e.g., Shurin, 2000; Tilman, 1997). Thus, recipient communities, whose species composition varied due to isolation, may have exhibited differential resistances to the establishment of species from different habitats.

Our results on species richness and beta diversity suggest differential extinctions in isolated communities. Dispersal from the same habitat source caused colonization and rescue effects, whereas dispersal from a different habitat likely introduced new species through mass effects. Additional evidence came from comparisons between source‐recipient pairs. Overall beta diversity for pairs from the same habitat source (dissimilarity between A0–Aa) remained stable over time, but its components showed opposite trends. Turnover decreased as colonists from isolated communities established in recipient communities, while nestedness increased as the isolated source community lost species, and the recipient maintained its species richness. Pairs from different habitat sources (B0–Ab) presented similar patterns, although initial overall beta diversity and turnover were much higher.

Our study demonstrates that isolated communities lose species over time, with differential species losses across communities driving increasing compositional divergence. Dispersal from similar habitats can halt both species richness decline and community divergence, while dispersal from different habitats not only prevents local diversity loss but can also drive species accumulation. These findings reveal the critical role of dispersal in balancing extinction and colonization dynamics, reshaping community structure through rescue effects, mass effects, and stochastic turnover. By integrating robust metacommunity theory with a carefully designed outdoor experiment, our work advances the understanding of how dispersal shapes biodiversity patterns in fragmented landscapes. It provides a cornerstone for predicting ecological resilience in a changing world and guiding efforts to maintain connectivity (e.g., corridors, permeable landscapes) among local communities.

## CONFLICT OF INTEREST STATEMENT

The authors declare no conflicts of interest.

## Supporting information


Appendix S1.

## Data Availability

Data and code (Melo, 2025) are available at Figshare at https://doi.org/10.6084/m9.figshare.28651985.v1.
