# Supplementary material for: An experimental test on the effects of dispersal from different habitat sources on community structure
Source: Ecology. 2025 Nov 17;106(11):e70256. doi: 10.1002/ecy.70256 (PMC12623123; doi:10.1002/ecy.70256)
Supplement: Supplementary file 1 — Appendix S1. [file ECY-106-e70256-s001.pdf]

Ecology **Appendix S1**

An experimental test on the effects of dispersal from different habitat sources on community structure

Gustavo L. Villarreal, Fernanda A. S. Cassemiro, Priscilla Carvalho, Luis M. Bini, Jascieli C. Bortolini, Amanda C. F. Queiroz, Wilson M. Leão-Neto, Roger P. Mormul, Ludgero C. G. Vieira, João C. Nabout, Fabricio B. Teresa, Maisa C. Vieira, Karine B. Machado, Tadeu Siqueira & Adriano S. Melo

The experimental area was established at Anápolis (Goiás, Brazil) at 16°22'44" S and 48°56'45" W, with nearest pond located 550 m away. The area was fenced to impede access to terrestrial vertebrates, and the floor was covered with coarse sand to avoid grass establishment (Fig. S1). The experimental area includes 80 tanks and a block of 40 (6 rows of 8 tanks) was used in the experiment (Fig. S2). Tanks were buried to prevent excessive warming and covered with semitransparent fabric (1.4 x 0.7 mm mesh) to impede visiting birds and mosquito proliferation.

Nutrients levels (NO_3^- and $PO4^{3-}$) were previously analyzed in lakes A (0.6 and 0.05 mg/L, respectively), lake B (0.9 and < 0.01 mg/L), and the well (0.1 and < 0.01 mg/L). Because the nutrient concentrations in the well were lower, nutrients were added to the tanks during setup to match the levels observed in the lakes. Tanks filled with lake A water received 41 ml of $NaNO_3$ and 3.5 ml of KH_2PO_4, whereas tanks with lake B water received 60 ml of $NaNO_3$ and 0.70 ml of KH_2PO_4. Nutrients were also added following the first and third dispersal events.

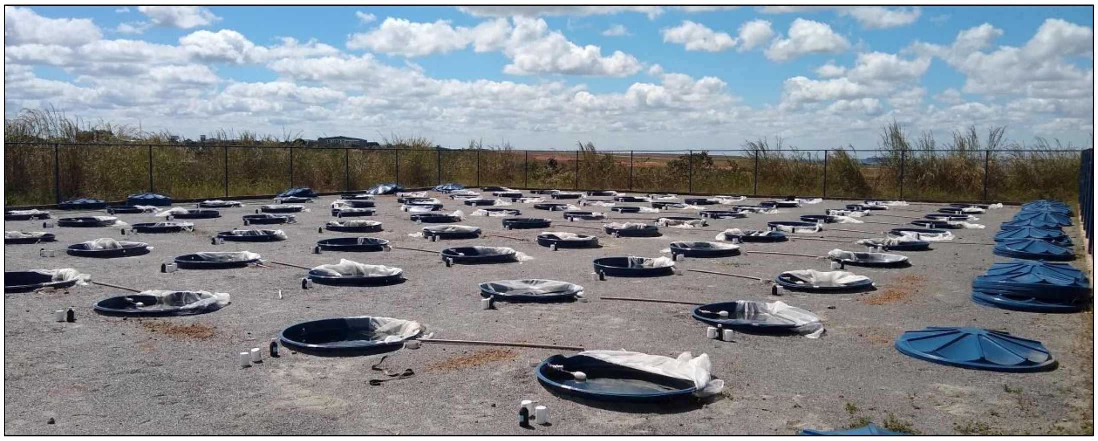

Figure S1. Experimental area at Anápolis, Brazil. Water tanks of 500 L were buried to prevent excessive warming and covered with semitransparent fabric to avoid terrestrial animals. Tanks were spaced 1.5 m apart. The experimental area includes 80 tanks, and 40 were used in the experiment. Photo credit: Adriano S. Melo.

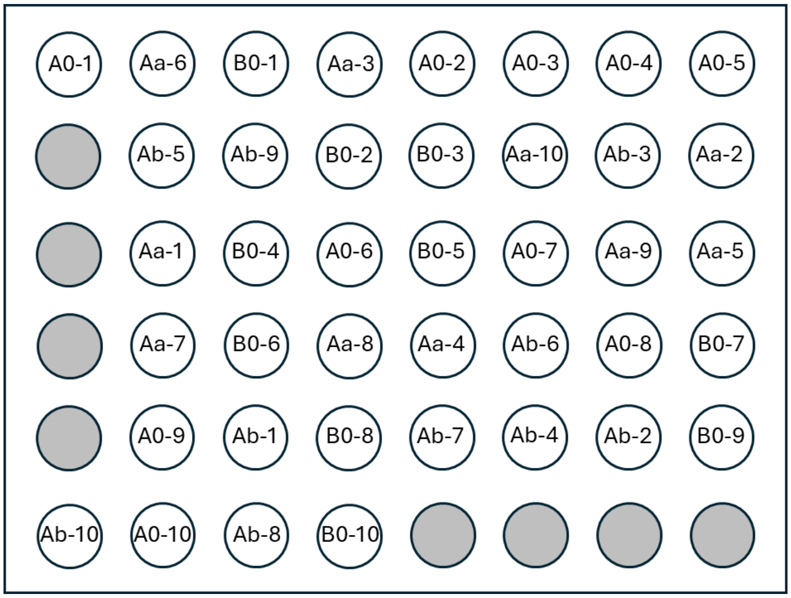

Figure S2. Water tanks were randomly assigned to treatments. Tanks in treatments A0 and B0 were filled with water of lakes A and B, respectively. Tanks Aa and Ab were filled with water from lake A, but received dispersers from A0 and B0 tanks, respectively. Ten tanks were

randomly chosen for treatment A0 and numbered consecutively from left to right, and from top to bottom. The same procedure was used for treatment B0. Tanks in treatments Aa and Ab were also randomly chosen, but were numbered according to the order in which they were drawn. The experimental design included pairs, with each pair defined by a number on the label. Thus, a donating tank, such as A0-1, provided disperser to its paired receiving tank, Aa-1. Likewise, B0-5 donated dispersers to Ab-5.
